# Strain driven fast osseointegration of implants

**DOI:** 10.1186/1746-160X-1-6

**Published:** 2005-09-01

**Authors:** Ulrich Joos, Andre Büchter, Hans-Peter Wiesmann, Ulrich Meyer

**Affiliations:** 1 Department of Cranio-Maxillofacial Surgery, University of Münster, Waldeyerstraße 30, D-48129 Münster, Germany

## Abstract

**Background:**

Although the bone's capability of dental implant osseointegration has clinically been utilised as early as in the Gallo-Roman population, the specific mechanisms for the emergence and maintenance of peri-implant bone under functional load have not been identified. Here we show that under immediate loading of specially designed dental implants with masticatory loads, osseointegration is rapidly achieved.

**Methods:**

We examined the bone reaction around non- and immediately loaded dental implants inserted in the mandible of mature minipigs during the presently assumed time for osseointegration. We used threaded conical titanium implants containing a titanium2+ oxide surface, allowing direct bone contact after insertion. The external geometry was designed according to finite element analysis: the calculation showed that physiological amplitudes of strain (500–3,000 ustrain) generated through mastication were homogenously distributed in peri-implant bone. The strain-energy density (SED) rate under assessment of a 1 Hz loading cycle was 150 Jm-3 s-1, peak dislocations were lower then nm.

**Results:**

Bone was in direct contact to the implant surface (bone/implant contact rate 90%) from day one of implant insertion, as quantified by undecalcified histological sections. This effect was substantiated by ultrastructural analysis of intimate osteoblast attachment and mature collagen mineralisation at the titanium surface. We detected no loss in the intimate bone/implant bond during the experimental period of either control or experimental animals, indicating that immediate load had no adverse effect on bone structure in peri-implant bone.

**Conclusion:**

In terms of clinical relevance, the load related bone reaction at the implant interface may in combination with substrate effects be responsible for an immediate osseointegration state.

## Findings

Although the bone's capability of dental implant osseointegration has clinically been utilised as early as in the Gallo-Roman population [1], the specific mechanisms for the emergence and maintenance of peri-implant bone under functional load have not been identified. Here we show that under immediate loading of specially designed dental implants with masticatory loads, osseointegration is rapidly achieved. As the osseointegration state is much faster reached than commonly assumed, osseointegration is a strain dependant highly dynamic process.

Osseointegration is defined as a direct and stable anchorage of an implant by the formation of bony tissue without growth of fibrous tissue at the bone-implant interface [2]. A defining feature of osseointegration is that osteoblasts and mineralized matrix contacts the implant surface even when loads are applied. A common perception is that several weeks must be given to achieve implant osseointegration.

**Figure 1:**
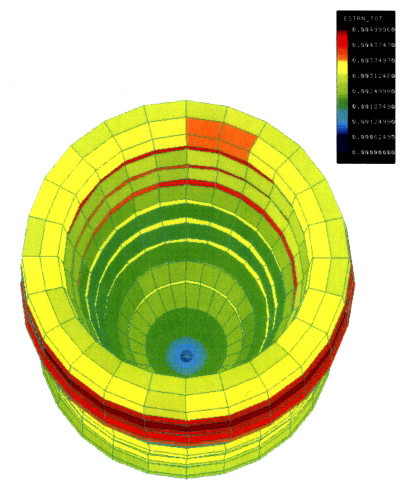
Biomechanics and biology of implant osseointegration.

We have departed from this time related hypothesis by proposing that only minimal time (for example few hours, the time that is necessary for osteoblast adhesion on artificial substrates [3]) is required for osseointegration when the peri-implant tissue receives an optimal mechanical environment. We examined the bone reaction around non- and immediately loaded dental implants inserted in the mandible of mature minipigs during the presently assumed time for osseointegration (approved by the Animal Ethics Committee of the University of Münster under the reference number G 90/99). We used threaded conical titanium implants containing a titanium^2+ ^oxide surface, allowing direct bone contact after insertion. The external geometry was designed according to finite element analysis: the calculation showed that physiological amplitudes of strain (500–3,000μstrain) generated through mastication were homogenously distributed in peri-implant bone (Figure 1). The strain-energy density (SED) rate [4] under assessment of a 1 Hz loading cycle was 150 Jm^-3 ^s^-1^, peak dislocations were lower then nm. Eigth male Göttinger minipigs, 14 to 16 months of age with an average body weight of 35 kg, were used in this study. At day 3, day 7 and 28 animals were sacrificed with an overdose of T61 given intravenously.

**Figure 2:**
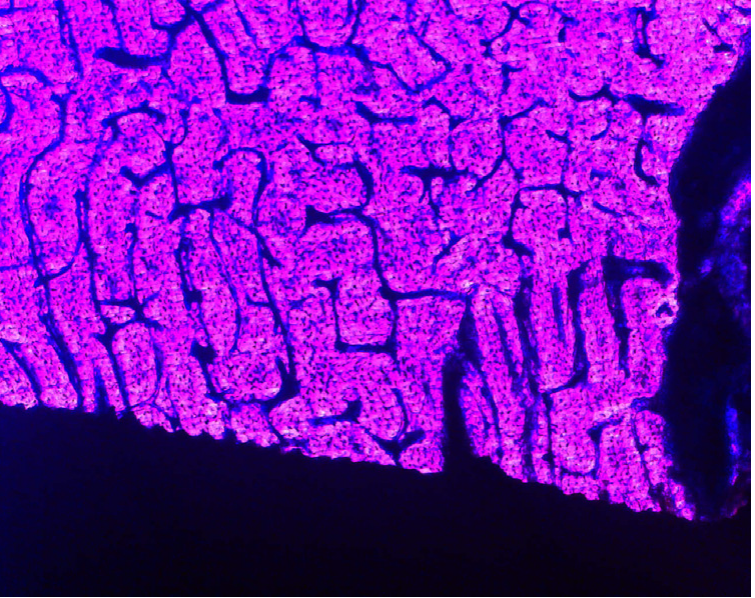
Finite element model of strain distribution in peri-implant bone. Bone strains do not exceed physiological values, bone dislocations are between 0 and 50 nm.

**Figure 3:**
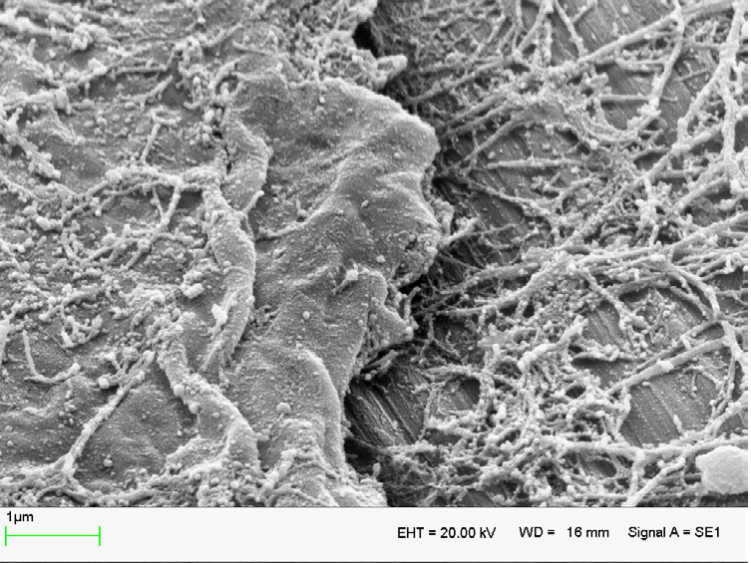
Histological picture of implant containing bone one day after insertion. Direct contact between bone and the implant is visible in the scanning electron micrographs.

**Figure 4:**
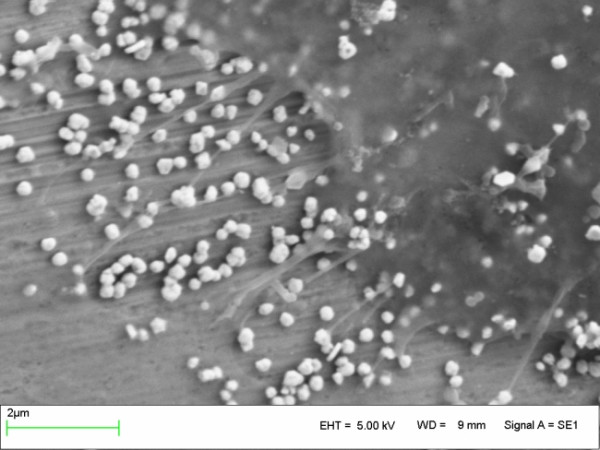
Immuno-scanning electron microscopy of intimate osteoblast adhesion at the titanium surface by fibronectin mediated focal adhesions (fractured specimens, one day under loading).

Bone was in direct contact to the implant surface (bone/implant contact rate 90%) from day one of implant insertion, as quantified by undecalcified histological sections (Figure 2). This effect was substantiated by ultrastructural analysis of intimate osteoblast attachment (Figure 3) and mature collagen mineralisation at the titanium surface. We detected no loss in the intimate bone/implant bond during the experimental period of either control or experimental animals, indicating that immediate load had no adverse effect on bone structure in peri-implant bone (Figure 4).

Bone response on an implant surface depends on the reaction of cells and matrix towards the material surface as well as to the mechanical constraints in the vincinity of the implant. The maintenance of bone and its adaptation to external loads is based on a complex strain driven regulatory process of cells and matrix components [5,6]. Outside-in mechanical tension exert direct effects on cell behaviour by activating biochemical signalling pathways and regulating gene expression through focal adhesions [7]. Frost [8] provided a paradigm for the mechanical control of cellular bone modelling, the process whereby bone is laid down onto surfaces without necessarily preceded by resorption. Recent investigations have indicated that the strain related bone modelling process is also regulative for bone tissue formation in healing tissue [9].

Using an atomic force microscope, a molecular mechanistic origin for the remarkably fast recovery of toughness after bone deformation was found, when deformation of less then 50 nm at the surface of multivalent ions (as in the case of Ti-oxide) is present [10]. Our understanding of osseointegration theorises that bone strengthening responds to a highly specific mechanical design. Even if long-term osseointegrated implants show what seems to be similar bone tissue reactions, osseointegration might be able to be achieved more rapidly than otherwise observed. Screw type titanium implants, as used in dental implantology, have in contrast to orthopaedic implants not only been convincingly shown very good clinical long-term success [11], but were also successful when load transfer is immediately present as seen in traumatology. In terms of clinical relevance, the load related bone reaction at the implant interface may in combination with substrate effects be responsible for an immediate osseointegration state.

## Competing interests

The author(s) declare that they have no competing interests.

## Authors' contributions

UJ designed the study, searched the database, extracted the data. AB helped with the study design and analysis. HPW had analysis the histological probes and UJ developed the implant design.
